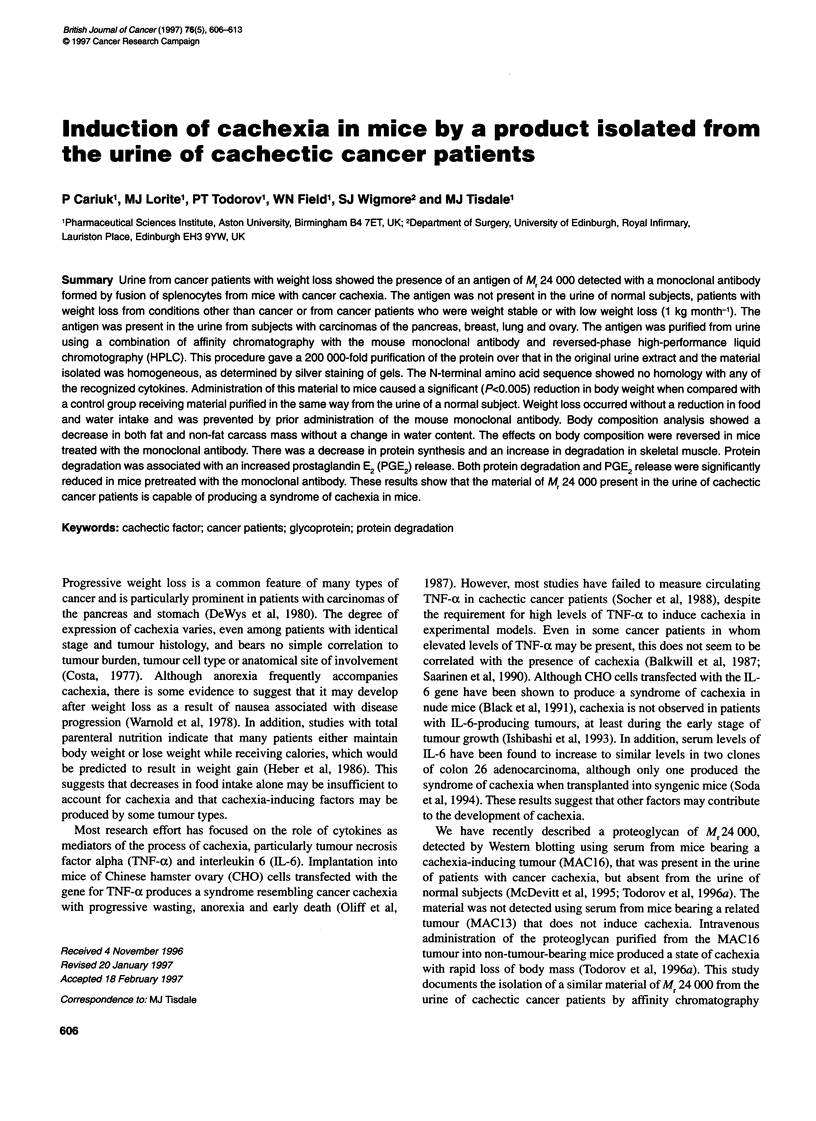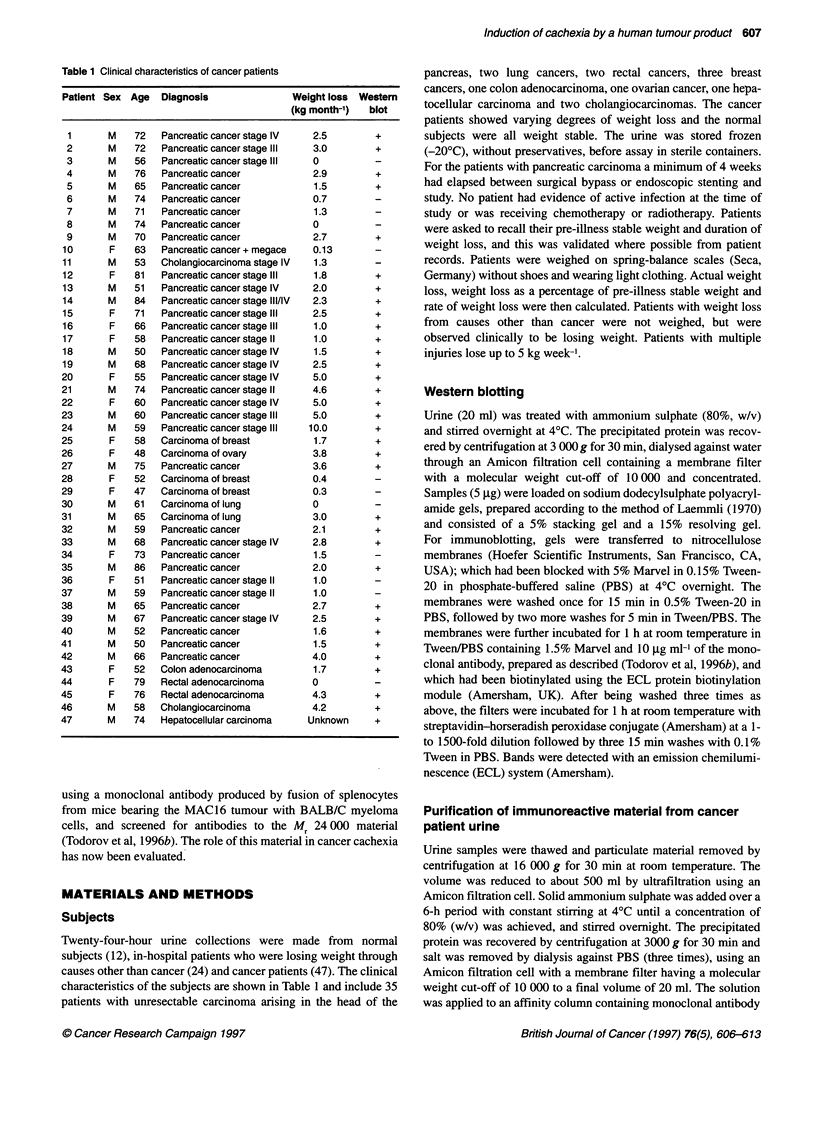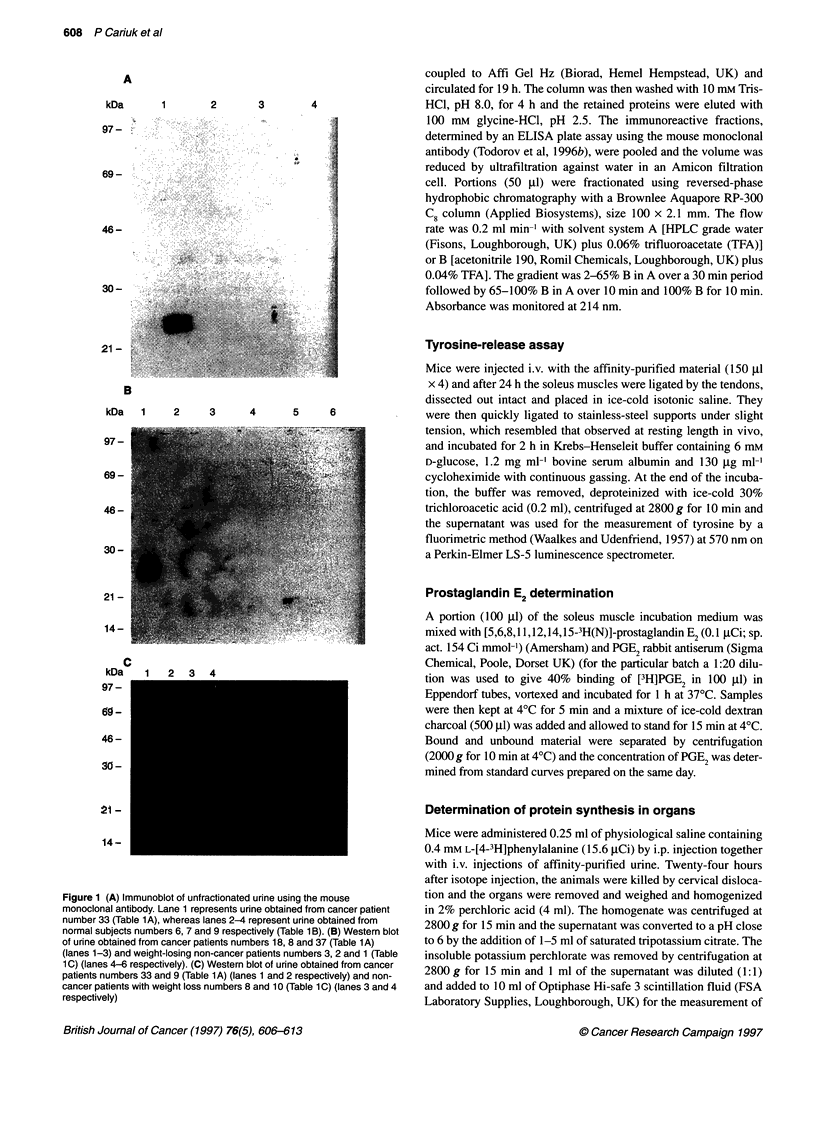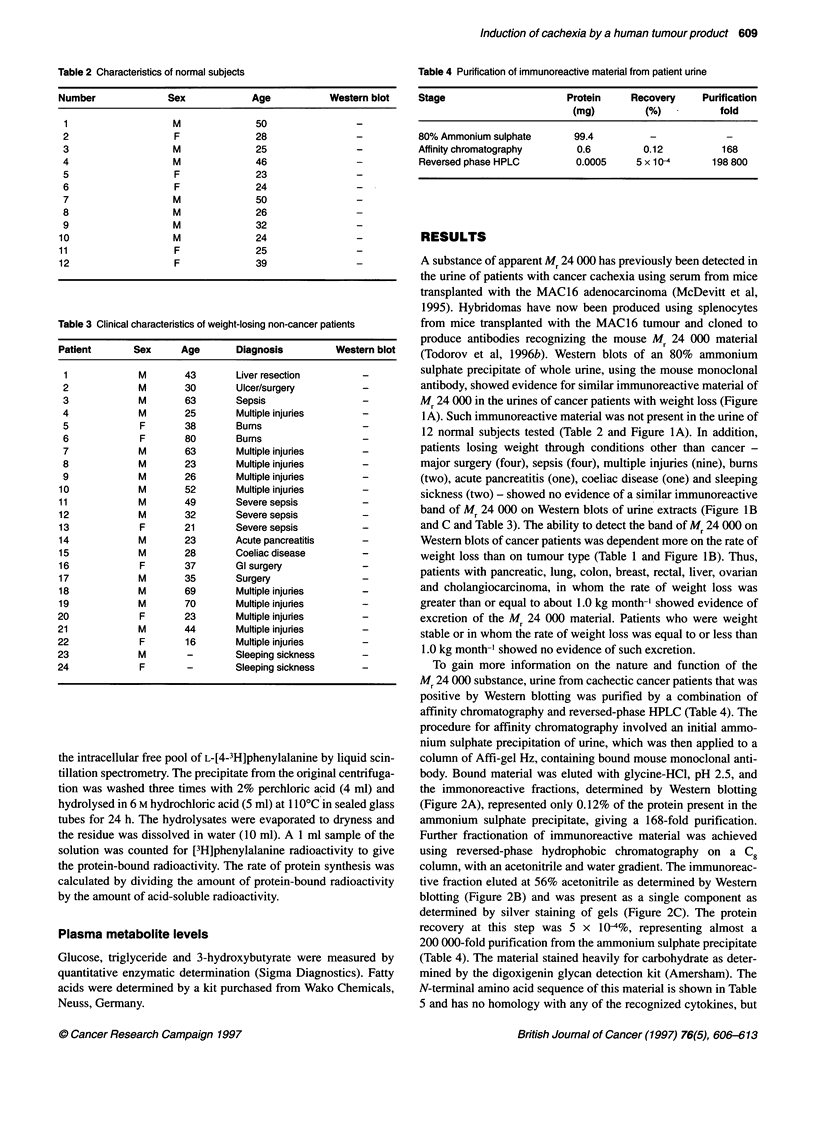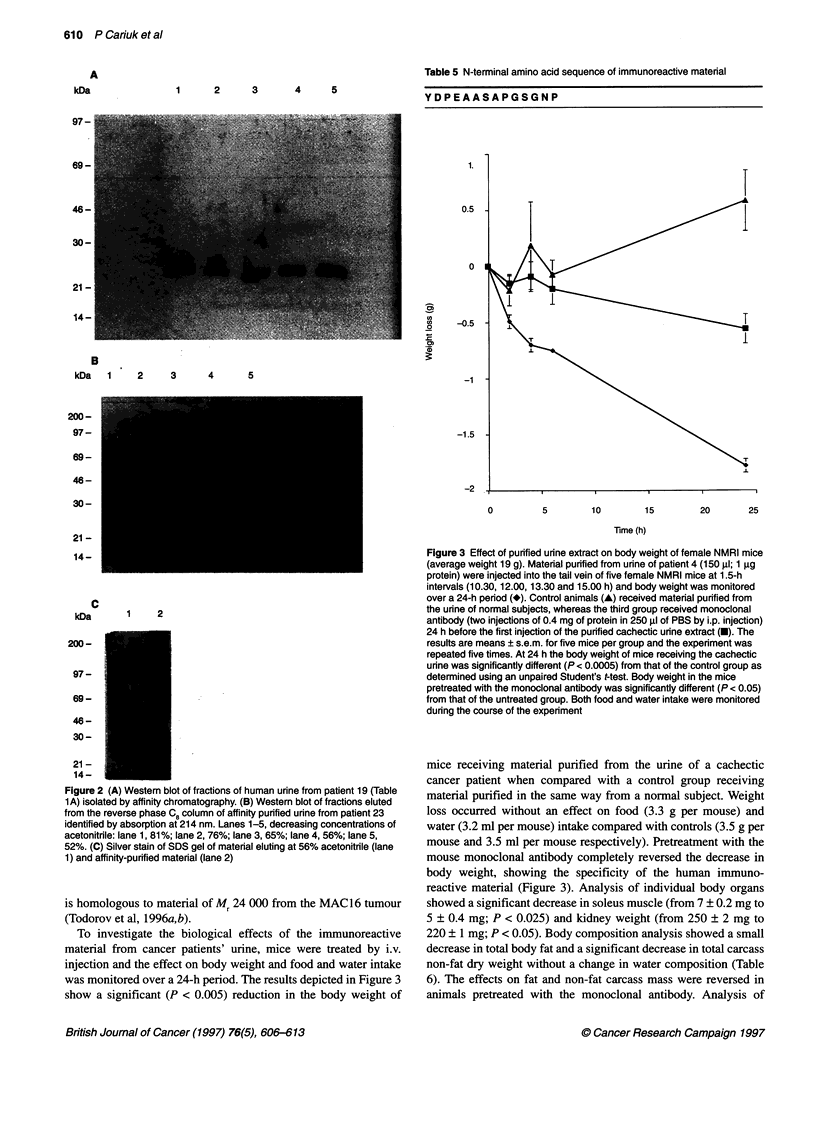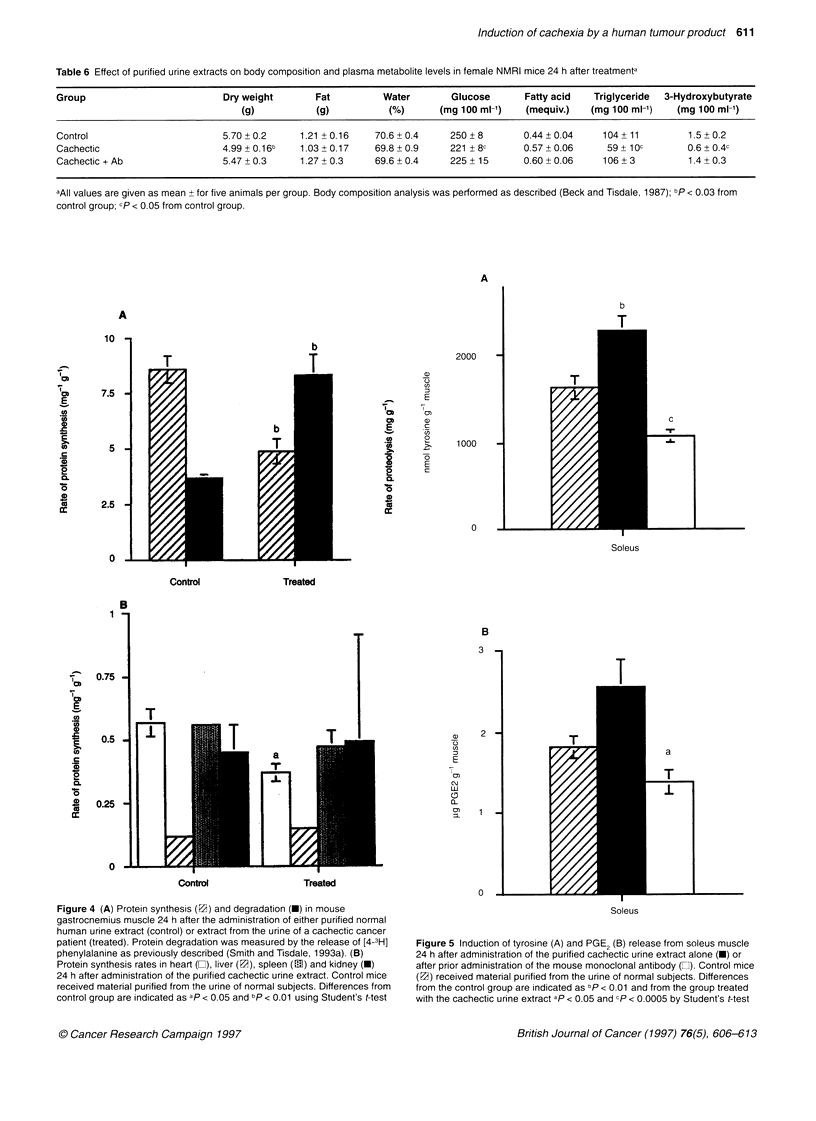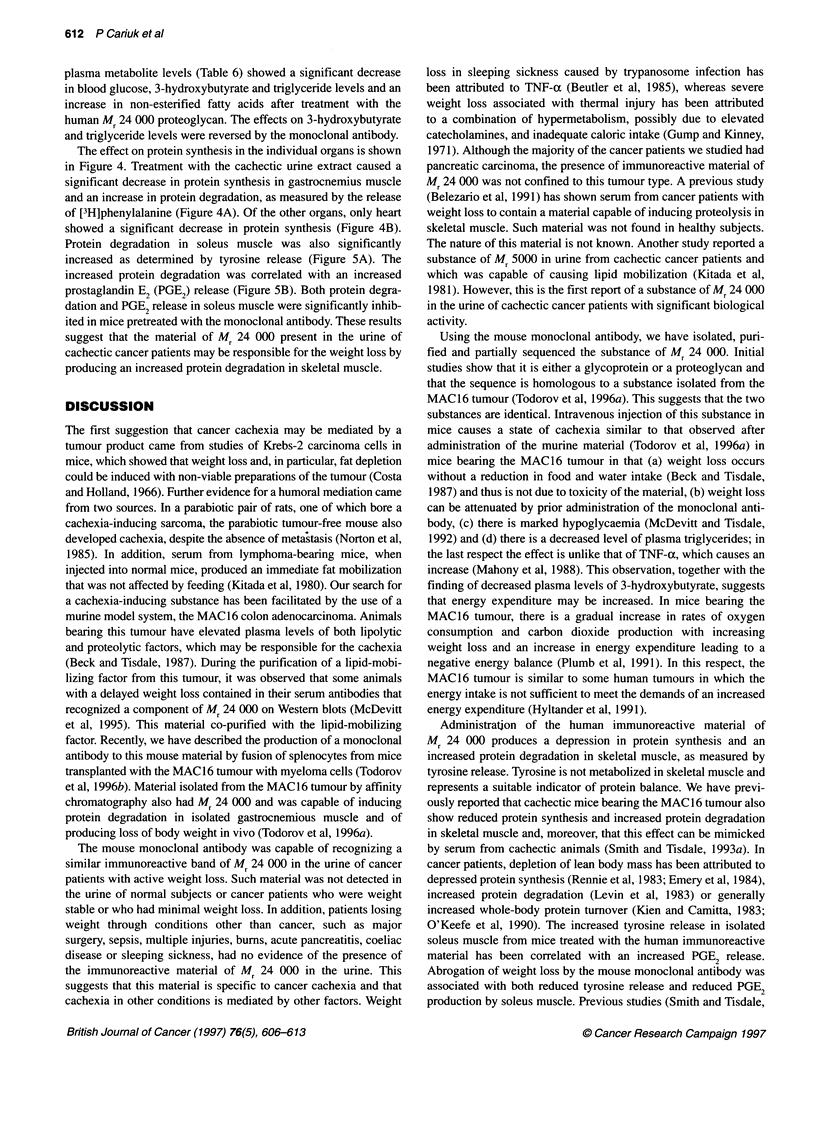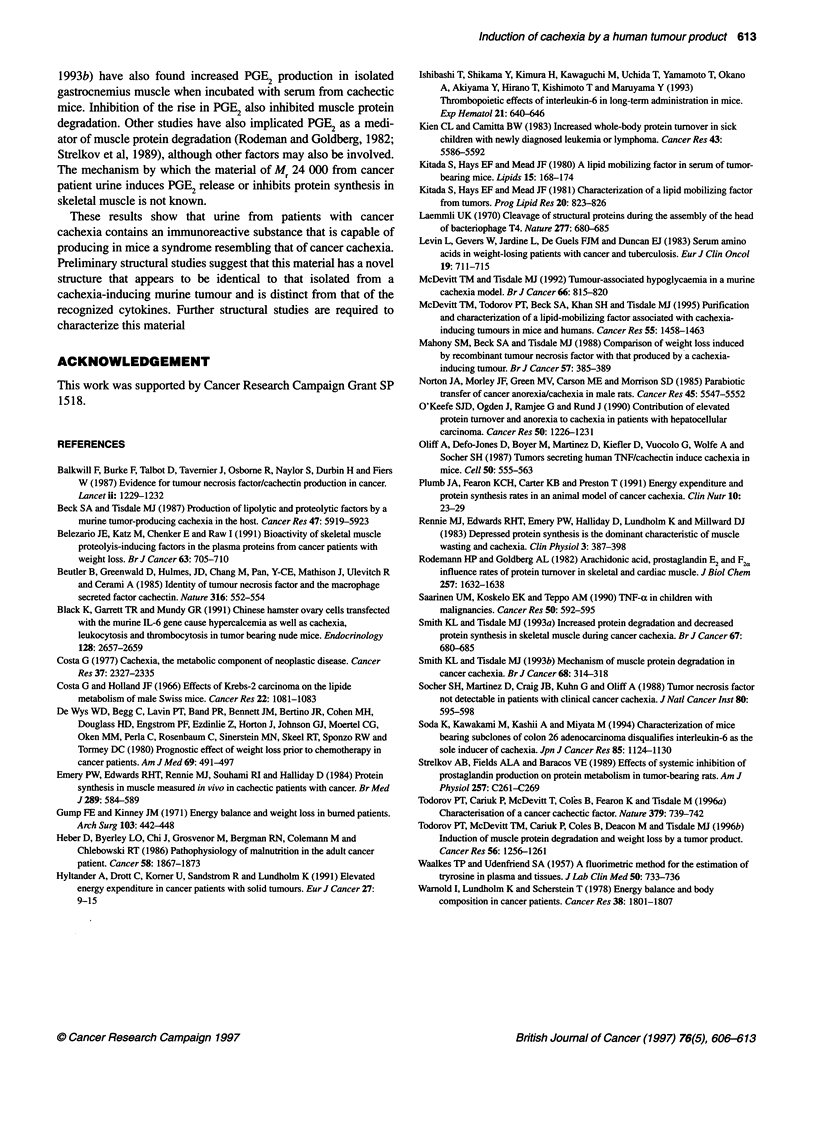# Induction of cachexia in mice by a product isolated from the urine of cachectic cancer patients.

**DOI:** 10.1038/bjc.1997.433

**Published:** 1997

**Authors:** P. Cariuk, M. J. Lorite, P. T. Todorov, W. N. Field, S. J. Wigmore, M. J. Tisdale

**Affiliations:** Pharmaceutical Sciences Institute, Aston University, Birmingham, UK.

## Abstract

**Images:**


British Journal of Cancer (1997) 76(5), 606–613
© 1997 Cancer Research Campaign

### Induction of cachexia in mice by a product isolated from the urine of cachectic cancer patients

**P Cariuk[1], MJ Lorite[1], PT Todorov[1], WN Field[1], SJ Wigmore[2] and MJ Tisdale[1]**

[1]Pharmaceutical Sciences Institute, Aston University, Birmingham B4 7ET, UK; [2]Department of Surgery, University of Edinburgh, Royal Infirmary, Lauriston Place, Edinburgh EH3 9YW, UK

**Summary** Urine from cancer patients with weight loss showed the presence of an antigen of $M_r$ 24 000 detected with a monoclonal antibody formed by fusion of splenocytes from mice with cancer cachexia. The antigen was not present in the urine of normal subjects, patients with weight loss from conditions other than cancer or from cancer patients who were weight stable or with low weight loss (1 kg month$^{-1}$). The antigen was present in the urine from subjects with carcinomas of the pancreas, breast, lung and ovary. The antigen was purified from urine using a combination of affinity chromatography with the mouse monoclonal antibody and reversed-phase high-performance liquid chromotography (HPLC). This procedure gave a 200 000-fold purification of the protein over that in the original urine extract and the material isolated was homogeneous, as determined by silver staining of gels. The N-terminal amino acid sequence showed no homology with any of the recognized cytokines. Administration of this material to mice caused a significant ($P$<0.005) reduction in body weight when compared with a control group receiving material purified in the same way from the urine of a normal subject. Weight loss occurred without a reduction in food and water intake and was prevented by prior administration of the mouse monoclonal antibody. Body composition analysis showed a decrease in both fat and non-fat carcass mass without a change in water content. The effects on body composition were reversed in mice treated with the monoclonal antibody. There was a decrease in protein synthesis and an increase in degradation in skeletal muscle. Protein degradation was associated with an increased prostaglandin $E_2$ ($PGE_2$) release. Both protein degradation and $PGE_2$ release were significantly reduced in mice pretreated with the monoclonal antibody. These results show that the material of $M_r$ 24 000 present in the urine of cachectic cancer patients is capable of producing a syndrome of cachexia in mice.

**Keywords:** cachectic factor; cancer patients; glycoprotein; protein degradation

Progressive weight loss is a common feature of many types of cancer and is particularly prominent in patients with carcinomas of the pancreas and stomach (DeWys et al, 1980). The degree of expression of cachexia varies, even among patients with identical stage and tumour histology, and bears no simple correlation to tumour burden, tumour cell type or anatomical site of involvement (Costa, 1977). Although anorexia frequently accompanies cachexia, there is some evidence to suggest that it may develop after weight loss as a result of nausea associated with disease progression (Warnold et al, 1978). In addition, studies with total parenteral nutrition indicate that many patients either maintain body weight or lose weight while receiving calories, which would be predicted to result in weight gain (Heber et al, 1986). This suggests that decreases in food intake alone may be insufficient to account for cachexia and that cachexia-inducing factors may be produced by some tumour types.

Most research effort has focused on the role of cytokines as mediators of the process of cachexia, particularly tumour necrosis factor alpha (TNF-α) and interleukin 6 (IL-6). Implantation into mice of Chinese hamster ovary (CHO) cells transfected with the gene for TNF-α produces a syndrome resembling cancer cachexia with progressive wasting, anorexia and early death (Oliff et al, 1987). However, most studies have failed to measure circulating TNF-α in cachectic cancer patients (Socher et al, 1988), despite the requirement for high levels of TNF-α to induce cachexia in experimental models. Even in some cancer patients in whom elevated levels of TNF-α may be present, this does not seem to be correlated with the presence of cachexia (Balkwill et al, 1987; Saarinen et al, 1990). Although CHO cells transfected with the IL-6 gene have been shown to produce a syndrome of cachexia in nude mice (Black et al, 1991), cachexia is not observed in patients with IL-6-producing tumours, at least during the early stage of tumour growth (Ishibashi et al, 1993). In addition, serum levels of IL-6 have been found to increase to similar levels in two clones of colon 26 adenocarcinoma, although only one produced the syndrome of cachexia when transplanted into syngenic mice (Soda et al, 1994). These results suggest that other factors may contribute to the development of cachexia.

We have recently described a proteoglycan of $M_r$ 24 000, detected by Western blotting using serum from mice bearing a cachexia-inducing tumour (MAC16), that was present in the urine of patients with cancer cachexia, but absent from the urine of normal subjects (McDevitt et al, 1995; Todorov et al, 1996*a*). The material was not detected using serum from mice bearing a related tumour (MAC13) that does not induce cachexia. Intravenous administration of the proteoglycan purified from the MAC16 tumour into non-tumour-bearing mice produced a state of cachexia with rapid loss of body mass (Todorov et al, 1996*a*). This study documents the isolation of a similar material of $M_r$ 24 000 from the urine of cachectic cancer patients by affinity chromatography

*Received 4 November 1996*
*Revised 20 January 1997*
*Accepted 18 February 1997*

*Correspondence to:* MJ Tisdale

**Table 1** Clinical characteristics of cancer patients

| Patient | Sex | Age | Diagnosis | Weight loss (kg month$^{-1}$) | Western blot |
|---|---|---|---|---|---|
| 1 | M | 72 | Pancreatic cancer stage IV | 2.5 | + |
| 2 | M | 72 | Pancreatic cancer stage III | 3.0 | + |
| 3 | M | 56 | Pancreatic cancer stage III | 0 | – |
| 4 | M | 76 | Pancreatic cancer | 2.9 | + |
| 5 | M | 65 | Pancreatic cancer | 1.5 | + |
| 6 | M | 74 | Pancreatic cancer | 0.7 | – |
| 7 | M | 71 | Pancreatic cancer | 1.3 | – |
| 8 | M | 74 | Pancreatic cancer | 0 | – |
| 9 | M | 70 | Pancreatic cancer | 2.7 | + |
| 10 | F | 63 | Pancreatic cancer + megace | 0.13 | – |
| 11 | M | 53 | Cholangiocarcinoma stage IV | 1.3 | – |
| 12 | F | 81 | Pancreatic cancer stage III | 1.8 | + |
| 13 | M | 51 | Pancreatic cancer stage IV | 2.0 | + |
| 14 | M | 84 | Pancreatic cancer stage III/IV | 2.3 | + |
| 15 | F | 71 | Pancreatic cancer stage III | 2.5 | + |
| 16 | F | 66 | Pancreatic cancer stage III | 1.0 | + |
| 17 | F | 58 | Pancreatic cancer stage II | 1.0 | + |
| 18 | M | 50 | Pancreatic cancer stage IV | 1.5 | + |
| 19 | M | 68 | Pancreatic cancer stage IV | 2.5 | + |
| 20 | F | 55 | Pancreatic cancer stage IV | 5.0 | + |
| 21 | M | 74 | Pancreatic cancer stage II | 4.6 | + |
| 22 | F | 60 | Pancreatic cancer stage IV | 5.0 | + |
| 23 | M | 60 | Pancreatic cancer stage III | 5.0 | + |
| 24 | M | 59 | Pancreatic cancer stage III | 10.0 | + |
| 25 | F | 58 | Carcinoma of breast | 1.7 | + |
| 26 | F | 48 | Carcinoma of ovary | 3.8 | + |
| 27 | M | 75 | Pancreatic cancer | 3.6 | + |
| 28 | F | 52 | Carcinoma of breast | 0.4 | – |
| 29 | F | 47 | Carcinoma of breast | 0.3 | – |
| 30 | M | 61 | Carcinoma of lung | 0 | – |
| 31 | M | 65 | Carcinoma of lung | 3.0 | + |
| 32 | M | 59 | Pancreatic cancer | 2.1 | + |
| 33 | M | 68 | Pancreatic cancer stage IV | 2.8 | + |
| 34 | F | 73 | Pancreatic cancer | 1.5 | – |
| 35 | M | 86 | Pancreatic cancer | 2.0 | + |
| 36 | F | 51 | Pancreatic cancer stage II | 1.0 | – |
| 37 | M | 59 | Pancreatic cancer stage II | 1.0 | – |
| 38 | M | 65 | Pancreatic cancer | 2.7 | + |
| 39 | M | 67 | Pancreatic cancer stage IV | 2.5 | + |
| 40 | M | 52 | Pancreatic cancer | 1.6 | + |
| 41 | M | 50 | Pancreatic cancer | 1.5 | + |
| 42 | M | 66 | Pancreatic cancer | 4.0 | + |
| 43 | F | 52 | Colon adenocarcinoma | 1.7 | + |
| 44 | F | 79 | Rectal adenocarcinoma | 0 | – |
| 45 | F | 76 | Rectal adenocarcinoma | 4.3 | + |
| 46 | M | 58 | Cholangiocarcinoma | 4.2 | + |
| 47 | M | 74 | Hepatocellular carcinoma | Unknown | + |

using a monoclonal antibody produced by fusion of splenocytes from mice bearing the MAC16 tumour with BALB/C myeloma cells, and screened for antibodies to the $M_r$ 24 000 material (Todorov et al, 1996*b*). The role of this material in cancer cachexia has now been evaluated.

### MATERIALS AND METHODS

### Subjects

Twenty-four-hour urine collections were made from normal subjects (12), in-hospital patients who were losing weight through causes other than cancer (24) and cancer patients (47). The clinical characteristics of the subjects are shown in Table 1 and include 35 patients with unresectable carcinoma arising in the head of the pancreas, two lung cancers, two rectal cancers, three breast cancers, one colon adenocarcinoma, one ovarian cancer, one hepatocellular carcinoma and two cholangiocarcinomas. The cancer patients showed varying degrees of weight loss and the normal subjects were all weight stable. The urine was stored frozen (–20°C), without preservatives, before assay in sterile containers. For the patients with pancreatic carcinoma a minimum of 4 weeks had elapsed between surgical bypass or endoscopic stenting and study. No patient had evidence of active infection at the time of study or was receiving chemotherapy or radiotherapy. Patients were asked to recall their pre-illness stable weight and duration of weight loss, and this was validated where possible from patient records. Patients were weighed on spring-balance scales (Seca, Germany) without shoes and wearing light clothing. Actual weight loss, weight loss as a percentage of pre-illness stable weight and rate of weight loss were then calculated. Patients with weight loss from causes other than cancer were not weighed, but were observed clinically to be losing weight. Patients with multiple injuries lose up to 5 kg week$^{-1}$.

### Western blotting

Urine (20 ml) was treated with ammonium sulphate (80%, w/v) and stirred overnight at 4°C. The precipitated protein was recovered by centrifugation at 3 000 ***g*** for 30 min, dialysed against water through an Amicon filtration cell containing a membrane filter with a molecular weight cut-off of 10 000 and concentrated. Samples (5 μg) were loaded on sodium dodecylsulphate polyacrylamide gels, prepared according to the method of Laemmli (1970) and consisted of a 5% stacking gel and a 15% resolving gel. For immunoblotting, gels were transferred to nitrocellulose membranes (Hoefer Scientific Instruments, San Francisco, CA, USA); which had been blocked with 5% Marvel in 0.15% Tween-20 in phosphate-buffered saline (PBS) at 4°C overnight. The membranes were washed once for 15 min in 0.5% Tween-20 in PBS, followed by two more washes for 5 min in Tween/PBS. The membranes were further incubated for 1 h at room temperature in Tween/PBS containing 1.5% Marvel and 10 μg ml$^{-1}$ of the monoclonal antibody, prepared as described (Todorov et al, 1996*b*), and which had been biotinylated using the ECL protein biotinylation module (Amersham, UK). After being washed three times as above, the filters were incubated for 1 h at room temperature with streptavidin–horseradish peroxidase conjugate (Amersham) at a 1- to 1500-fold dilution followed by three 15 min washes with 0.1% Tween in PBS. Bands were detected with an emission chemiluminescence (ECL) system (Amersham).

### Purification of immunoreactive material from cancer patient urine

Urine samples were thawed and particulate material removed by centrifugation at 16 000 *g* for 30 min at room temperature. The volume was reduced to about 500 ml by ultrafiltration using an Amicon filtration cell. Solid ammonium sulphate was added over a 6-h period with constant stirring at 4°C until a concentration of 80% (w/v) was achieved, and stirred overnight. The precipitated protein was recovered by centrifugation at 3000 *g* for 30 min and salt was removed by dialysis against PBS (three times), using an Amicon filtration cell with a membrane filter having a molecular weight cut-off of 10 000 to a final volume of 20 ml. The solution was applied to an affinity column containing monoclonal antibody

© Cancer Research Campaign 1997 

**Figure 1** (**A**) Immunoblot of unfractionated urine using the mouse monoclonal antibody. Lane 1 represents urine obtained from cancer patient number 33 (Table 1A), whereas lanes 2–4 represent urine obtained from normal subjects numbers 6, 7 and 9 respectively (Table 1B). (**B**) Western blot of urine obtained from cancer patients numbers 18, 8 and 37 (Table 1A) (lanes 1–3) and weight-losing non-cancer patients numbers 3, 2 and 1 (Table 1C) (lanes 4–6 respectively). (**C**) Western blot of urine obtained from cancer patients numbers 33 and 9 (Table 1A) (lanes 1 and 2 respectively) and non-cancer patients with weight loss numbers 8 and 10 (Table 1C) (lanes 3 and 4 respectively)

coupled to Affi Gel Hz (Biorad, Hemel Hempstead, UK) and circulated for 19 h. The column was then washed with 10 mM Tris-HCl, pH 8.0, for 4 h and the retained proteins were eluted with 100 mM glycine-HCl, pH 2.5. The immunoreactive fractions, determined by an ELISA plate assay using the mouse monoclonal antibody (Todorov et al, 1996*b*), were pooled and the volume was reduced by ultrafiltration against water in an Amicon filtration cell. Portions (50 μl) were fractionated using reversed-phase hydrophobic chromatography with a Brownlee Aquapore RP-300 $C_8$ column (Applied Biosystems), size 100 × 2.1 mm. The flow rate was 0.2 ml $min^{-1}$ with solvent system A [HPLC grade water (Fisons, Loughborough, UK) plus 0.06% trifluoroacetate (TFA)] or B [acetonitrile 190, Romil Chemicals, Loughborough, UK) plus 0.04% TFA]. The gradient was 2–65% B in A over a 30 min period followed by 65–100% B in A over 10 min and 100% B for 10 min. Absorbance was monitored at 214 nm.

### Tyrosine-release assay

Mice were injected i.v. with the affinity-purified material (150 μl × 4) and after 24 h the soleus muscles were ligated by the tendons, dissected out intact and placed in ice-cold isotonic saline. They were then quickly ligated to stainless-steel supports under slight tension, which resembled that observed at resting length in vivo, and incubated for 2 h in Krebs–Henseleit buffer containing 6 mM D-glucose, 1.2 mg $ml^{-1}$ bovine serum albumin and 130 μg $ml^{-1}$ cycloheximide with continuous gassing. At the end of the incubation, the buffer was removed, deproteinized with ice-cold 30% trichloroacetic acid (0.2 ml), centrifuged at 2800 ***g*** for 10 min and the supernatant was used for the measurement of tyrosine by a fluorimetric method (Waalkes and Udenfriend, 1957) at 570 nm on a Perkin-Elmer LS-5 luminescence spectrometer.

### Prostaglandin $E_2$ determination

A portion (100 μl) of the soleus muscle incubation medium was mixed with [5,6,8,11,12,14,15-$^3$H(N)]-prostaglandin $E_2$ (0.1 μCi; sp. act. 154 Ci $mmol^{-1}$) (Amersham) and $PGE_2$ rabbit antiserum (Sigma Chemical, Poole, Dorset UK) (for the particular batch a 1:20 dilution was used to give 40% binding of [$^3$H]$PGE_2$ in 100 μl) in Eppendorf tubes, vortexed and incubated for 1 h at 37°C. Samples were then kept at 4°C for 5 min and a mixture of ice-cold dextran charcoal (500 μl) was added and allowed to stand for 15 min at 4°C. Bound and unbound material were separated by centrifugation (2000 ***g*** for 10 min at 4°C) and the concentration of $PGE_2$ was determined from standard curves prepared on the same day.

### Determination of protein synthesis in organs

Mice were administered 0.25 ml of physiological saline containing 0.4 mM L-[4-$^3$H]phenylalanine (15.6 μCi) by i.p. injection together with i.v. injections of affinity-purified urine. Twenty-four hours after isotope injection, the animals were killed by cervical dislocation and the organs were removed and weighed and homogenized in 2% perchloric acid (4 ml). The homogenate was centrifuged at 2800 ***g*** for 15 min and the supernatant was converted to a pH close to 6 by the addition of 1–5 ml of saturated tripotassium citrate. The insoluble potassium perchlorate was removed by centrifugation at 2800 ***g*** for 15 min and 1 ml of the supernatant was diluted (1:1) and added to 10 ml of Optiphase Hi-safe 3 scintillation fluid (FSA Laboratory Supplies, Loughborough, UK) for the measurement of

© Cancer Research Campaign 1997

**Table 2** Characteristics of normal subjects

| Number | Sex | Age | Western blot |
|---|---|---|---|
| 1 | M | 50 | – |
| 2 | F | 28 | – |
| 3 | M | 25 | – |
| 4 | M | 46 | – |
| 5 | F | 23 | – |
| 6 | F | 24 | – |
| 7 | M | 50 | – |
| 8 | M | 26 | – |
| 9 | M | 32 | – |
| 10 | M | 24 | – |
| 11 | F | 25 | – |
| 12 | F | 39 | – |

**Table 3** Clinical characteristics of weight-losing non-cancer patients

| Patient | Sex | Age | Diagnosis | Western blot |
|---|---|---|---|---|
| 1 | M | 43 | Liver resection | – |
| 2 | M | 30 | Ulcer/surgery | – |
| 3 | M | 63 | Sepsis | – |
| 4 | M | 25 | Multiple injuries | – |
| 5 | F | 38 | Burns | – |
| 6 | F | 80 | Burns | – |
| 7 | M | 63 | Multiple injuries | – |
| 8 | M | 23 | Multiple injuries | – |
| 9 | M | 26 | Multiple injuries | – |
| 10 | M | 52 | Multiple injuries | – |
| 11 | M | 49 | Severe sepsis | – |
| 12 | M | 32 | Severe sepsis | – |
| 13 | F | 21 | Severe sepsis | – |
| 14 | M | 23 | Acute pancreatitis | – |
| 15 | M | 28 | Coeliac disease | – |
| 16 | F | 37 | GI surgery | – |
| 17 | M | 35 | Surgery | – |
| 18 | M | 69 | Multiple injuries | – |
| 19 | M | 70 | Multiple injuries | – |
| 20 | F | 23 | Multiple injuries | – |
| 21 | M | 44 | Multiple injuries | – |
| 22 | F | 16 | Multiple injuries | – |
| 23 | M | – | Sleeping sickness | – |
| 24 | F | – | Sleeping sickness | – |

the intracellular free pool of L-[4-$^3$H]phenylalanine by liquid scintillation spectrometry. The precipitate from the original centrifugation was washed three times with 2% perchloric acid (4 ml) and hydrolysed in 6 M hydrochloric acid (5 ml) at 110°C in sealed glass tubes for 24 h. The hydrolysates were evaporated to dryness and the residue was dissolved in water (10 ml). A 1 ml sample of the solution was counted for [$^3$H]phenylalanine radioactivity to give the protein-bound radioactivity. The rate of protein synthesis was calculated by dividing the amount of protein-bound radioactivity by the amount of acid-soluble radioactivity.

### Plasma metabolite levels

Glucose, triglyceride and 3-hydroxybutyrate were measured by quantitative enzymatic determination (Sigma Diagnostics). Fatty acids were determined by a kit purchased from Wako Chemicals, Neuss, Germany.

**Table 4** Purification of immunoreactive material from patient urine

| Stage | Protein (mg) | Recovery (%) | Purification fold |
|---|---|---|---|
| 80% Ammonium sulphate | 99.4 | – | – |
| Affinity chromatography | 0.6 | 0.12 | 168 |
| Reversed phase HPLC | 0.0005 | $5 \times 10^{-4}$ | 198 800 |

### RESULTS

A substance of apparent $M_r$ 24 000 has previously been detected in the urine of patients with cancer cachexia using serum from mice transplanted with the MAC16 adenocarcinoma (McDevitt et al, 1995). Hybridomas have now been produced using splenocytes from mice transplanted with the MAC16 tumour and cloned to produce antibodies recognizing the mouse $M_r$ 24 000 material (Todorov et al, 1996*b*). Western blots of an 80% ammonium sulphate precipitate of whole urine, using the mouse monoclonal antibody, showed evidence for similar immunoreactive material of $M_r$ 24 000 in the urines of cancer patients with weight loss (Figure 1A). Such immunoreactive material was not present in the urine of 12 normal subjects tested (Table 2 and Figure 1A). In addition, patients losing weight through conditions other than cancer – major surgery (four), sepsis (four), multiple injuries (nine), burns (two), acute pancreatitis (one), coeliac disease (one) and sleeping sickness (two) – showed no evidence of a similar immunoreactive band of $M_r$ 24 000 on Western blots of urine extracts (Figure 1B and C and Table 3). The ability to detect the band of $M_r$ 24 000 on Western blots of cancer patients was dependent more on the rate of weight loss than on tumour type (Table 1 and Figure 1B). Thus, patients with pancreatic, lung, colon, breast, rectal, liver, ovarian and cholangiocarcinoma, in whom the rate of weight loss was greater than or equal to about 1.0 kg month$^{-1}$ showed evidence of excretion of the $M_r$ 24 000 material. Patients who were weight stable or in whom the rate of weight loss was equal to or less than 1.0 kg month$^{-1}$ showed no evidence of such excretion.

To gain more information on the nature and function of the $M_r$ 24 000 substance, urine from cachectic cancer patients that was positive by Western blotting was purified by a combination of affinity chromatography and reversed-phase HPLC (Table 4). The procedure for affinity chromatography involved an initial ammonium sulphate precipitation of urine, which was then applied to a column of Affi-gel Hz, containing bound mouse monoclonal antibody. Bound material was eluted with glycine-HCl, pH 2.5, and the immonoreactive fractions, determined by Western blotting (Figure 2A), represented only 0.12% of the protein present in the ammonium sulphate precipitate, giving a 168-fold purification. Further fractionation of immunoreactive material was achieved using reversed-phase hydrophobic chromatography on a $C_8$ column, with an acetonitrile and water gradient. The immunoreactive fraction eluted at 56% acetonitrile as determined by Western blotting (Figure 2B) and was present as a single component as determined by silver staining of gels (Figure 2C). The protein recovery at this step was $5 \times 10^{-4}$%, representing almost a 200 000-fold purification from the ammonium sulphate precipitate (Table 4). The material stained heavily for carbohydrate as determined by the digoxigenin glycan detection kit (Amersham). The *N*-terminal amino acid sequence of this material is shown in Table 5 and has no homology with any of the recognized cytokines, but

© Cancer Research Campaign 1997

**Figure 2** (**A**) Western blot of fractions of human urine from patient 19 (Table 1A) isolated by affinity chromatography. (**B**) Western blot of fractions eluted from the reverse phase $C_8$ column of affinity purified urine from patient 23 identified by absorption at 214 nm. Lanes 1–5, decreasing concentrations of acetonitrile: lane 1, 81%; lane 2, 76%; lane 3, 65%; lane 4, 56%; lane 5, 52%. (**C**) Silver stain of SDS gel of material eluting at 56% acetonitrile (lane 1) and affinity-purified material (lane 2)

is homologous to material of $M_r$ 24 000 from the MAC16 tumour (Todorov et al, 1996*a*,*b*).

To investigate the biological effects of the immunoreactive material from cancer patients' urine, mice were treated by i.v. injection and the effect on body weight and food and water intake was monitored over a 24-h period. The results depicted in Figure 3 show a significant ($P$ < 0.005) reduction in the body weight of mice receiving material purified from the urine of a cachectic cancer patient when compared with a control group receiving material purified in the same way from a normal subject. Weight loss occurred without an effect on food (3.3 g per mouse) and water (3.2 ml per mouse) intake compared with controls (3.5 g per mouse and 3.5 ml per mouse respectively). Pretreatment with the mouse monoclonal antibody completely reversed the decrease in body weight, showing the specificity of the human immunoreactive material (Figure 3). Analysis of individual body organs showed a significant decrease in soleus muscle (from 7 ± 0.2 mg to 5 ± 0.4 mg; $P$ < 0.025) and kidney weight (from 250 ± 2 mg to 220 ± 1 mg; $P$ < 0.05). Body composition analysis showed a small decrease in total body fat and a significant decrease in total carcass non-fat dry weight without a change in water composition (Table 6). The effects on fat and non-fat carcass mass were reversed in animals pretreated with the monoclonal antibody. Analysis of

**Table 5** N-terminal amino acid sequence of immunoreactive material

| |
|---|
| **YDPEAASAPGSGNP** |

**Figure 3** Effect of purified urine extract on body weight of female NMRI mice (average weight 19 g). Material purified from urine of patient 4 (150 µl; 1 µg protein) were injected into the tail vein of five female NMRI mice at 1.5-h intervals (10.30, 12.00, 13.30 and 15.00 h) and body weight was monitored over a 24-h period (◆). Control animals (▲) received material purified from the urine of normal subjects, whereas the third group received monoclonal antibody (two injections of 0.4 mg of protein in 250 µl of PBS by i.p. injection) 24 h before the first injection of the purified cachectic urine extract (■). The results are means ± s.e.m. for five mice per group and the experiment was repeated five times. At 24 h the body weight of mice receiving the cachectic urine was significantly different ($P$ < 0.0005) from that of the control group as determined using an unpaired Student's $t$-test. Body weight in the mice pretreated with the monoclonal antibody was significantly different ($P$ < 0.05) from that of the untreated group. Both food and water intake were monitored during the course of the experiment

© *Cancer Research Campaign 1997*

**Table 6** Effect of purified urine extracts on body composition and plasma metabolite levels in female NMRI mice 24 h after treatment[a]

| Group | Dry weight (g) | Fat (g) | Water (%) | Glucose (mg 100 ml$^{-1}$) | Fatty acid (mequiv.) | Triglyceride (mg 100 ml$^{-1}$) | 3-Hydroxybutyrate (mg 100 ml$^{-1}$) |
|---|---|---|---|---|---|---|---|
| Control | 5.70 ± 0.2 | 1.21 ± 0.16 | 70.6 ± 0.4 | 250 ± 8 | 0.44 ± 0.04 | 104 ± 11 | 1.5 ± 0.2 |
| Cachectic | 4.99 ± 0.16[b] | 1.03 ± 0.17 | 69.8 ± 0.9 | 221 ± 8[c] | 0.57 ± 0.06 | 59 ± 10[c] | 0.6 ± 0.4[c] |
| Cachectic + Ab | 5.47 ± 0.3 | 1.27 ± 0.3 | 69.6 ± 0.4 | 225 ± 15 | 0.60 ± 0.06 | 106 ± 3 | 1.4 ± 0.3 |

[a]All values are given as mean ± for five animals per group. Body composition analysis was performed as described (Beck and Tisdale, 1987); [b]$P < 0.03$ from control group; [c]$P < 0.05$ from control group.

**Figure 4** (**A**) Protein synthesis (▨) and degradation (■) in mouse gastrocnemius muscle 24 h after the administration of either purified normal human urine extract (control) or extract from the urine of a cachectic cancer patient (treated). Protein degradation was measured by the release of [4-$^3$H] phenylalanine as previously described (Smith and Tisdale, 1993a). (**B**) Protein synthesis rates in heart (□), liver (▨), spleen (▩) and kidney (■) 24 h after administration of the purified cachectic urine extract. Control mice received material purified from the urine of normal subjects. Differences from control group are indicated as [a]$P < 0.05$ and [b]$P < 0.01$ using Student's *t*-test

**Figure 5** Induction of tyrosine (A) and PGE$_2$ (B) release from soleus muscle 24 h after administration of the purified cachectic urine extract alone (■) or after prior administration of the mouse monoclonal antibody (□). Control mice (▨) received material purified from the urine of normal subjects. Differences from the control group are indicated as [b]$P < 0.01$ and from the group treated with the cachectic urine extract [a]$P < 0.05$ and [c]$P < 0.0005$ by Student's *t*-test plasma metabolite levels (Table 6) showed a significant decrease in blood glucose, 3-hydroxybutyrate and triglyceride levels and an increase in non-esterified fatty acids after treatment with the human $M_r$ 24 000 proteoglycan. The effects on 3-hydroxybutyrate and triglyceride levels were reversed by the monoclonal antibody.

The effect on protein synthesis in the individual organs is shown in Figure 4. Treatment with the cachectic urine extract caused a significant decrease in protein synthesis in gastrocnemius muscle and an increase in protein degradation, as measured by the release of [$^3$H]phenylalanine (Figure 4A). Of the other organs, only heart showed a significant decrease in protein synthesis (Figure 4B). Protein degradation in soleus muscle was also significantly increased as determined by tyrosine release (Figure 5A). The increased protein degradation was correlated with an increased prostaglandin $E_2$ ($PGE_2$) release (Figure 5B). Both protein degradation and $PGE_2$ release in soleus muscle were significantly inhibited in mice pretreated with the monoclonal antibody. These results suggest that the material of $M_r$ 24 000 present in the urine of cachectic cancer patients may be responsible for the weight loss by producing an increased protein degradation in skeletal muscle.

### DISCUSSION

The first suggestion that cancer cachexia may be mediated by a tumour product came from studies of Krebs-2 carcinoma cells in mice, which showed that weight loss and, in particular, fat depletion could be induced with non-viable preparations of the tumour (Costa and Holland, 1966). Further evidence for a humoral mediation came from two sources. In a parabiotic pair of rats, one of which bore a cachexia-inducing sarcoma, the parabiotic tumour-free mouse also developed cachexia, despite the absence of metastasis (Norton et al, 1985). In addition, serum from lymphoma-bearing mice, when injected into normal mice, produced an immediate fat mobilization that was not affected by feeding (Kitada et al, 1980). Our search for a cachexia-inducing substance has been facilitated by the use of a murine model system, the MAC16 colon adenocarcinoma. Animals bearing this tumour have elevated plasma levels of both lipolytic and proteolytic factors, which may be responsible for the cachexia (Beck and Tisdale, 1987). During the purification of a lipid-mobilizing factor from this tumour, it was observed that some animals with a delayed weight loss contained in their serum antibodies that recognized a component of $M_r$ 24 000 on Western blots (McDevitt et al, 1995). This material co-purified with the lipid-mobilizing factor. Recently, we have described the production of a monoclonal antibody to this mouse material by fusion of splenocytes from mice transplanted with the MAC16 tumour with myeloma cells (Todorov et al, 1996*b*). Material isolated from the MAC16 tumour by affinity chromatography also had $M_r$ 24 000 and was capable of inducing protein degradation in isolated gastrocnemious muscle and of producing loss of body weight in vivo (Todorov et al, 1996*a*).

The mouse monoclonal antibody was capable of recognizing a similar immunoreactive band of $M_r$ 24 000 in the urine of cancer patients with active weight loss. Such material was not detected in the urine of normal subjects or cancer patients who were weight stable or who had minimal weight loss. In addition, patients losing weight through conditions other than cancer, such as major surgery, sepsis, multiple injuries, burns, acute pancreatitis, coeliac disease or sleeping sickness, had no evidence of the presence of the immunoreactive material of $M_r$ 24 000 in the urine. This suggests that this material is specific to cancer cachexia and that cachexia in other conditions is mediated by other factors. Weight loss in sleeping sickness caused by trypanosome infection has been attributed to TNF-α (Beutler et al, 1985), whereas severe weight loss associated with thermal injury has been attributed to a combination of hypermetabolism, possibly due to elevated catecholamines, and inadequate caloric intake (Gump and Kinney, 1971). Although the majority of the cancer patients we studied had pancreatic carcinoma, the presence of immunoreactive material of $M_r$ 24 000 was not confined to this tumour type. A previous study (Belezario et al, 1991) has shown serum from cancer patients with weight loss to contain a material capable of inducing proteolysis in skeletal muscle. Such material was not found in healthy subjects. The nature of this material is not known. Another study reported a substance of $M_r$ 5000 in urine from cachectic cancer patients and which was capable of causing lipid mobilization (Kitada et al, 1981). However, this is the first report of a substance of $M_r$ 24 000 in the urine of cachectic cancer patients with significant biological activity.

Using the mouse monoclonal antibody, we have isolated, purified and partially sequenced the substance of $M_r$ 24 000. Initial studies show that it is either a glycoprotein or a proteoglycan and that the sequence is homologous to a substance isolated from the MAC16 tumour (Todorov et al, 1996*a*). This suggests that the two substances are identical. Intravenous injection of this substance in mice causes a state of cachexia similar to that observed after administration of the murine material (Todorov et al, 1996*a*) in mice bearing the MAC16 tumour in that (a) weight loss occurs without a reduction in food and water intake (Beck and Tisdale, 1987) and thus is not due to toxicity of the material, (b) weight loss can be attenuated by prior administration of the monoclonal antibody, (c) there is marked hypoglycaemia (McDevitt and Tisdale, 1992) and (d) there is a decreased level of plasma triglycerides; in the last respect the effect is unlike that of TNF-α, which causes an increase (Mahony et al, 1988). This observation, together with the finding of decreased plasma levels of 3-hydroxybutyrate, suggests that energy expenditure may be increased. In mice bearing the MAC16 tumour, there is a gradual increase in rates of oxygen consumption and carbon dioxide production with increasing weight loss and an increase in energy expenditure leading to a negative energy balance (Plumb et al, 1991). In this respect, the MAC16 tumour is similar to some human tumours in which the energy intake is not sufficient to meet the demands of an increased energy expenditure (Hyltander et al, 1991).

Administration of the human immunoreactive material of $M_r$ 24 000 produces a depression in protein synthesis and an increased protein degradation in skeletal muscle, as measured by tyrosine release. Tyrosine is not metabolized in skeletal muscle and represents a suitable indicator of protein balance. We have previously reported that cachectic mice bearing the MAC16 tumour also show reduced protein synthesis and increased protein degradation in skeletal muscle and, moreover, that this effect can be mimicked by serum from cachectic animals (Smith and Tisdale, 1993*a*). In cancer patients, depletion of lean body mass has been attributed to depressed protein synthesis (Rennie et al, 1983; Emery et al, 1984), increased protein degradation (Levin et al, 1983) or generally increased whole-body protein turnover (Kien and Camitta, 1983; O'Keefe et al, 1990). The increased tyrosine release in isolated soleus muscle from mice treated with the human immunoreactive material has been correlated with an increased $PGE_2$ release. Abrogation of weight loss by the mouse monoclonal antibody was associated with both reduced tyrosine release and reduced $PGE_2$ production by soleus muscle. Previous studies (Smith and Tisdale,

 © Cancer Research Campaign 1997

1993*b*) have also found increased $PGE_2$ production in isolated gastrocnemius muscle when incubated with serum from cachectic mice. Inhibition of the rise in $PGE_2$ also inhibited muscle protein degradation. Other studies have also implicated $PGE_2$ as a mediator of muscle protein degradation (Rodeman and Goldberg, 1982; Strelkov et al, 1989), although other factors may also be involved. The mechanism by which the material of $M_r$ 24 000 from cancer patient urine induces $PGE_2$ release or inhibits protein synthesis in skeletal muscle is not known.

These results show that urine from patients with cancer cachexia contains an immunoreactive substance that is capable of producing in mice a syndrome resembling that of cancer cachexia. Preliminary structural studies suggest that this material has a novel structure that appears to be identical to that isolated from a cachexia-inducing murine tumour and is distinct from that of the recognized cytokines. Further structural studies are required to characterize this material

### ACKNOWLEDGEMENT

This work was supported by Cancer Research Campaign Grant SP 1518.

### REFERENCES

Balkwill F, Burke F, Talbot D, Tavernier J, Osborne R, Naylor S, Durbin H and Fiers W (1987) Evidence for tumour necrosis factor/cachectin production in cancer. *Lancet* **ii**: 1229–1232

Beck SA and Tisdale MJ (1987) Production of lipolytic and proteolytic factors by a murine tumor-producing cachexia in the host. *Cancer Res* **47**: 5919–5923

Belezario JE, Katz M, Chenker E and Raw I (1991) Bioactivity of skeletal muscle proteolyis-inducing factors in the plasma proteins from cancer patients with weight loss. *Br J Cancer* **63**: 705–710

Beutler B, Greenwald D, Hulmes, JD, Chang M, Pan, Y-CE, Mathison J, Ulevitch R and Cerami A (1985) Identity of tumour necrosis factor and the macrophage secreted factor cachectin. *Nature* **316**: 552–554

Black K, Garrett TR and Mundy GR (1991) Chinese hamster ovary cells transfected with the murine IL-6 gene cause hypercalcemia as well as cachexia, leukocytosis and thrombocytosis in tumor bearing nude mice. *Endocrinology* **128**: 2657–2659

Costa G (1977) Cachexia, the metabolic component of neoplastic disease. *Cancer Res* **37**: 2327–2335

Costa G and Holland JF (1966) Effects of Krebs-2 carcinoma on the lipide metabolism of male Swiss mice. *Cancer Res* **22**: 1081–1083

De Wys WD, Begg C, Lavin PT, Band PR, Bennett JM, Bertino JR, Cohen MH, Douglass HD, Engstrom PF, Ezdinlie Z, Horton J, Johnson GJ, Moertel CG, Oken MM, Perla C, Rosenbaum C, Sinerstein MN, Skeel RT, Sponzo RW and Tormey DC (1980) Prognostic effect of weight loss prior to chemotherapy in cancer patients. *Am J Med* **69**: 491–497

Emery PW, Edwards RHT, Rennie MJ, Souhami RI and Halliday D (1984) Protein synthesis in muscle measured *in vivo* in cachectic patients with cancer. *Br Med J* **289**: 584–589

Gump FE and Kinney JM (1971) Energy balance and weight loss in burned patients. *Arch Surg* **103**: 442–448

Heber D, Byerley LO, Chi J, Grosvenor M, Bergman RN, Colemann M and Chlebowski RT (1986) Pathophysiology of malnutrition in the adult cancer patient. *Cancer* **58**: 1867–1873

Hyltander A, Drott C, Korner U, Sandstrom R and Lundholm K (1991) Elevated energy expenditure in cancer patients with solid tumours. *Eur J Cancer* **27**: 9–15

Ishibashi T, Shikama Y, Kimura H, Kawaguchi M, Uchida T, Yamamoto T, Okano A, Akiyama Y, Hirano T, Kishimoto T and Maruyama Y (1993) Thrombopoietic effects of interleukin-6 in long-term administration in mice. *Exp Hematol* **21**: 640–646

Kien CL and Camitta BW (1983) Increased whole-body protein turnover in sick children with newly diagnosed leukemia or lymphoma. *Cancer Res* **43**: 5586–5592

Kitada S, Hays EF and Mead JF (1980) A lipid mobilizing factor in serum of tumor-bearing mice. *Lipids* **15**: 168–174

Kitada S, Hays EF and Mead JF (1981) Characterization of a lipid mobilizing factor from tumors. *Prog Lipid Res* **20**: 823–826

Laemmli UK (1970) Cleavage of structural proteins during the assembly of the head of bacteriophage T4. *Nature* **277**: 680–685

Levin L, Gevers W, Jardine L, De Guels FJM and Duncan EJ (1983) Serum amino acids in weight-losing patients with cancer and tuberculosis. *Eur J Clin Oncol* **19**: 711–715

McDevitt TM and Tisdale MJ (1992) Tumour-associated hypoglycaemia in a murine cachexia model. *Br J Cancer* **66**: 815–820

McDevitt TM, Todorov PT, Beck SA, Khan SH and Tisdale MJ (1995) Purification and characterization of a lipid-mobilizing factor associated with cachexia-inducing tumours in mice and humans. *Cancer Res* **55**: 1458–1463

Mahony SM, Beck SA and Tisdale MJ (1988) Comparison of weight loss induced by recombinant tumour necrosis factor with that produced by a cachexia-inducing tumour. *Br J Cancer* **57**: 385–389

Norton JA, Morley JF, Green MV, Carson ME and Morrison SD (1985) Parabiotic transfer of cancer anorexia/cachexia in male rats. *Cancer Res* **45**: 5547–5552

O'Keefe SJD, Ogden J, Ramjee G and Rund J (1990) Contribution of elevated protein turnover and anorexia to cachexia in patients with hepatocellular carcinoma. *Cancer Res* **50**: 1226–1231

Oliff A, Defo-Jones D, Boyer M, Martinez D, Kiefler D, Vuocolo G, Wolfe A and Socher SH (1987) Tumors secreting human TNF/cachectin induce cachexia in mice. *Cell* **50**: 555–563

Plumb JA, Fearon KCH, Carter KB and Preston T (1991) Energy expenditure and protein synthesis rates in an animal model of cancer cachexia. *Clin Nutr* **10**: 23–29

Rennie MJ, Edwards RHT, Emery PW, Halliday D, Lundholm K and Millward DJ (1983) Depressed protein synthesis is the dominant characteristic of muscle wasting and cachexia. *Clin Physiol* **3**: 387–398

Rodemann HP and Goldberg AL (1982) Arachidonic acid, prostaglandin $E_2$ and $F_{2\alpha}$ influence rates of protein turnover in skeletal and cardiac muscle. *J Biol Chem* **257**: 1632–1638

Saarinen UM, Koskelo EK and Teppo AM (1990) TNF-α in children with malignancies. *Cancer Res* **50**: 592–595

Smith KL and Tisdale MJ (1993*a*) Increased protein degradation and decreased protein synthesis in skeletal muscle during cancer cachexia. *Br J Cancer* **67**: 680–685

Smith KL and Tisdale MJ (1993*b*) Mechanism of muscle protein degradation in cancer cachexia. *Br J Cancer* **68**: 314–318

Socher SH, Martinez D, Craig JB, Kuhn G and Oliff A (1988) Tumor necrosis factor not detectable in patients with clinical cancer cachexia. *J Natl Cancer Inst* **80**: 595–598

Soda K, Kawakami M, Kashii A and Miyata M (1994) Characterization of mice bearing subclones of colon 26 adenocarcinoma disqualifies interleukin-6 as the sole inducer of cachexia. *Jpn J Cancer Res* **85**: 1124–1130

Strelkov AB, Fields ALA and Baracos VE (1989) Effects of systemic inhibition of prostaglandin production on protein metabolism in tumor-bearing rats. *Am J Physiol* **257**: C261–C269

Todorov PT, Cariuk P, McDevitt T, Coles B, Fearon K and Tisdale M (1996*a*) Characterisation of a cancer cachectic factor. *Nature* **379**: 739–742

Todorov PT, McDevitt TM, Cariuk P, Coles B, Deacon M and Tisdale MJ (1996*b*) Induction of muscle protein degradation and weight loss by a tumor product. *Cancer Res* **56**: 1256–1261

Waalkes TP and Udenfriend SA (1957) A fluorimetric method for the estimation of tyrosine in plasma and tissues. *J Lab Clin Med* **50**: 733–736

Warnold I, Lundholm K and Scherstein T (1978) Energy balance and body composition in cancer patients. *Cancer Res* **38**: 1801–1807

© Cancer Research Campaign 1997